# Supplementary material for: High levels of intra-strain structural variation in Drosophila simulans X pericentric heterochromatin
Source: Genetics. 2023 Sep 28;225(4):iyad176. doi: 10.1093/genetics/iyad176 (PMC10697818; doi:10.1093/genetics/iyad176)
Supplement: iyad176_Supplementary_Data [file iyad176_supplementary_data.zip › Supplemental Material legends.docx]

SUPPLEMENTAL FIGURES

Supplemental Figure 1. Read coverage across the *X* chromosome assembly is not informative about the presence of structural variation within the isolates. We plotted read coverage (reads per million, RPM) across the *X* chromosomes in five public Illumina datasets for the strains *w^501^*, *w^XD1^*, *SR*, *ST8*, *C167.8*. The *w^501^* and *w^XD1^* strains may be polymorphic for multiple *X* pericentromere structural variants but libraries were prepared from pooled females. The breakpoints of possible structural rearrangements that may be present in the *w^501^* and *w^XD1^* libraries are not obvious from coverage plots on the assembled *X* chromosome. The breakpoint of the structural variant may be beyond the assembled region, but our ability to detect a breakpoint depends on the relative frequency of the different structural variants in the pool of individuals sequenced. We would need multiple biological replicates of each isolate and an assembly that extends through the pericentric heterochromatin to assess whether genomic approaches can detect the structural variation.
