# Supplementary figures and images for: High levels of intra-strain structural variation in Drosophila simulans X pericentric heterochromatin
Source: Genetics. 2023 Sep 28;225(4):iyad176. doi: 10.1093/genetics/iyad176 (PMC10697818; doi:10.1093/genetics/iyad176)

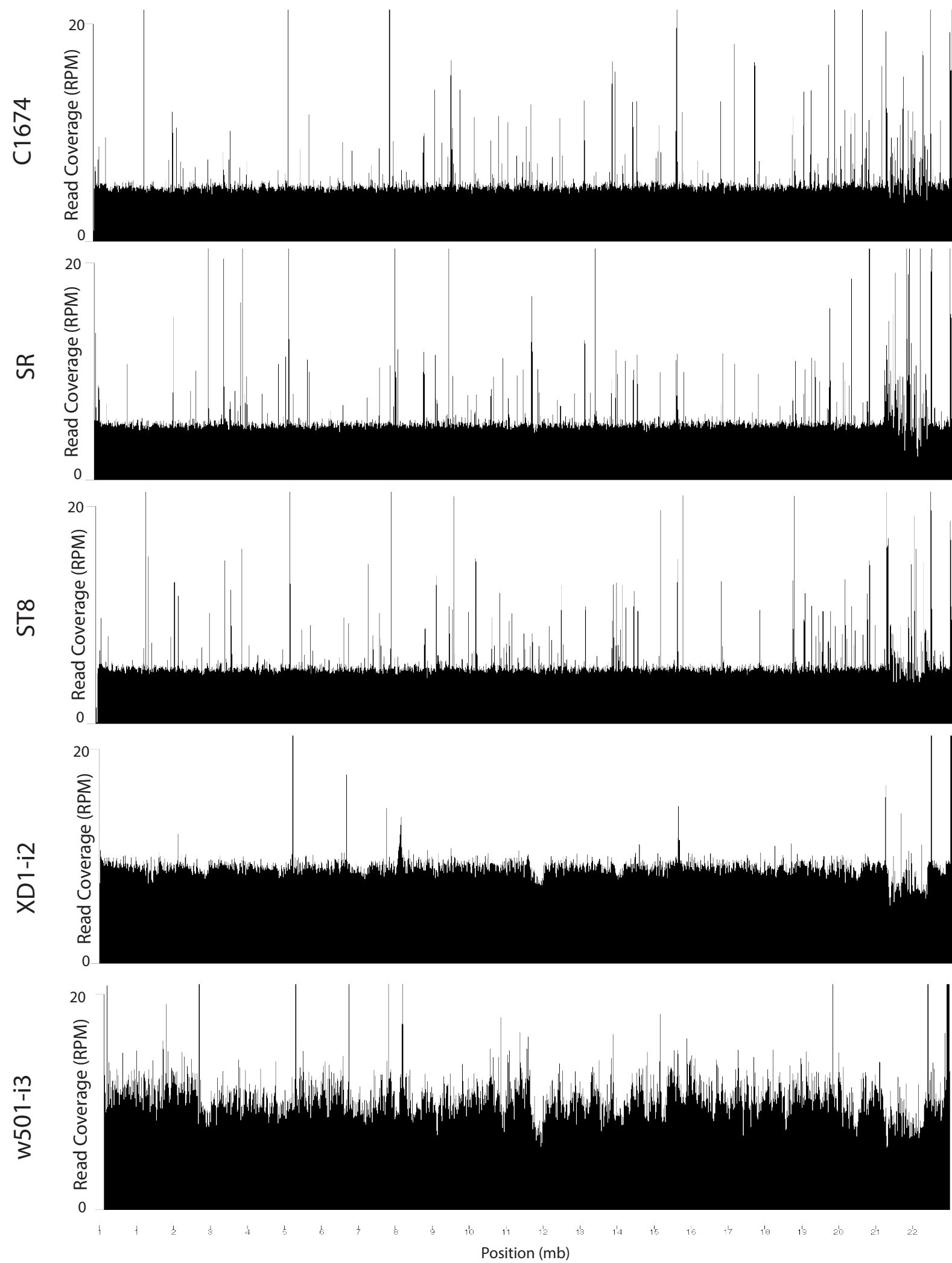

Supplement: iyad176_Supplementary_Data [file iyad176_supplementary_data.zip › FigureS1.pdf]
